# Intranasal Dexmedetomidine Versus Esketamine As Premedication for the Sedation of Pediatric Patients: A Systematic Review and Meta-Analysis

**DOI:** 10.7759/cureus.108763

**Published:** 2026-05-13

**Authors:** Lelian Ghaly, Ayat Al Nuaimi, Abdelrahman Ragab, Mohamed O Gamal, Mahmoud M Asida, Abdelrahman Sayed, Mohamad M Alemam, Manar Adel, Mohamed Hesham Gamal

**Affiliations:** 1 Neonatology, Queens Hospital Center, London, GBR; 2 Pediatrics, Northampton General Hospital, Northampton, GBR; 3 Plastic Surgery, Minia University Hospital, Minia, EGY; 4 Surgical Oncology, Nasser Institute Hospital for Research and Treatment, Cairo, EGY; 5 Pediatric Surgery, Minia University Hospital, Minia, EGY; 6 Urology, Minia University Hospital, Minia, EGY; 7 General Practice, Alexandria University, Alexandria, EGY; 8 Clinical Pharmacy, Tanta University, Gharbia, EGY; 9 Pharmacology and Therapeutics, Tanta University, Tanta, EGY

**Keywords:** dexmedetomidine, emergence delirium, esketamine, intranasal premedication, meta-analysis, pediatric anesthesia

## Abstract

Preoperative anxiety is a common problem in pediatric surgical patients and is a recognized predictor of emergence delirium (ED) and postoperative negative behavioral changes (PNBC). Intranasal premedication has emerged as a preferred route in children. Both dexmedetomidine and esketamine have demonstrated independent efficacy in this context. Their pharmacological complementarity has prompted growing interest in their combined use, yet no systematic review has comprehensively compared their efficacy and safety as monotherapies across pediatric procedural settings. This review aimed to evaluate and compare intranasal dexmedetomidine versus intranasal esketamine in pediatric patients. The study was conducted in accordance with the Cochrane Handbook and reported following Preferred Reporting Items for Systematic reviews and Meta-Analyses (PRISMA) guidelines. Five electronic databases were searched without date restriction. Risk of bias was assessed using the Cochrane RoB 2 tool for randomized controlled trials (RCTs) and the Newcastle-Ottawa Scale for the cohort study. Pooled risk ratios (RRs) and mean differences (MDs) with 95% confidence intervals were calculated using RevMan 5.4, with fixed- or random-effects models applied based on heterogeneity. The final analysis included five studies (four RCTs, one prospective cohort) with a combined sample of 481 pediatric participants. In elective surgical settings, dexmedetomidine at 2 µg/kg significantly reduced ED incidence compared to esketamine (RR 0.57 (0.38, 0.86); p=0.008) and was associated with significantly lower Pediatric Anesthesia Emergence Delirium (PAED) scale scores (MD -1.28 (-2.36, -0.20); p=0.02). Conversely, esketamine demonstrated significantly lower PAED scores in the bronchoscopy subgroup (MD 2.73 (1.84, 3.62); p<0.00001). Dexmedetomidine was also associated with significantly lower Face Legs Activity Cry Consolability (FLACC) pain scores in the emergency department subgroup (MD -3.25 (-5.64, -0.86); p=0.008). Regarding the primary endpoints, the overall pooled ED incidence did not differ significantly between agents (RR 0.77 (0.37, 1.62); p=0.49), nor did overall FLACC pain scores (MD -0.24 (-1.13, 0.65); p=0.60). Intranasal dexmedetomidine and esketamine demonstrate comparable overall efficacy and safety as pediatric premedication agents, with neither showing a significant pooled advantage over the other. However, their comparative performance is context- and dose-dependent, with dexmedetomidine at 2 µg/kg proving superior in elective surgical settings, and esketamine performing better in bronchoscopy procedures. These findings support individualized agent selection based on procedural context and dosing rather than a universal preference for either agent. Larger, standardized multicenter trials are needed to establish definitive evidence-based premedication recommendations across pediatric surgical settings.

## Introduction and background

Preoperative anxiety affects an estimated 50-70% of children undergoing surgical or procedural interventions, with the wide reported range reflecting variability in patient age, surgical type, anesthetic technique, and assessment instrument [1]. Preoperative anxiety has been linked to two clinically significant postoperative complications: emergence delirium (ED) and postoperative negative behavioral changes (PNBC). However, this relationship is complex and influenced by several factors, including patient age, baseline temperament, choice of inhalational agent, postoperative pain, and parental anxiety [1]. ED is an acute state of agitation and disorientation occurring during anesthesia recovery, with incidence rates of 30-80% under sevoflurane, the most widely used volatile agent in pediatric practice [2,3]. Children who experience ED carry a substantially elevated risk of developing PNBC, a cluster of maladaptive behavioral changes that may persist for weeks following discharge [4].

Intranasal premedication has emerged as a preferred approach in children, offering rapid drug absorption without the need for intravenous access or oral cooperation [5]. Two agents have attracted particular interest: dexmedetomidine [6], which is a selective α-2 adrenoceptor agonist, and esketamine [7], which acts through N-methyl-D-aspartate (NMDA) receptor antagonism to provide rapid dissociative sedation and analgesia while preserving airway reflexes. Both agents appear pharmacologically well-suited for combination use, as dexmedetomidine may counteract esketamine's cardiovascular stimulatory effects, while esketamine's faster onset may compensate for dexmedetomidine's slower onset [8].

Preliminary evidence suggests that intranasal co-administration of these agents yields superior sedation, lower ED incidence, and shorter emergence times compared to monotherapy, without increased adverse events [8,9]. However, no systematic review has yet comprehensively synthesized their comparative efficacy and safety across the full spectrum of pediatric procedural contexts. This systematic review and meta-analysis aimed to evaluate and compare the efficacy and safety of intranasal dexmedetomidine and intranasal esketamine as premedication agents in pediatric patients undergoing surgical or procedural interventions under general or local anesthesia.

## Review

Methods

This study followed the methodological framework outlined in the Cochrane Handbook for Systematic Reviews of Interventions [10], and manuscript reporting conformed to the Preferred Reporting Items for Systematic Reviews and Meta-Analyses (PRISMA) statement [11].

Search Strategy

A comprehensive systematic literature search was performed across multiple electronic databases to identify studies examining the role of Dexmedetomidine and Esketamine in pediatrics. The databases included PubMed, Web of Science (WOS), Scopus, Embase, and Cochrane Library. The search strategy employed a combination of controlled vocabulary (MeSH terms) and free-text keywords. Database-specific search terminology is presented in the Appendix.

Study Selection

Following comprehensive database searches, all retrieved records were imported into EndNote Software Version X-9. After duplicate entries were removed, the remaining records underwent a two-stage screening process: title and abstract screening, followed by full-text assessment. During the first stage, two independent reviewers evaluated all records against predefined inclusion and exclusion criteria, and any discrepancies were resolved through discussion and consensus.

We included studies that met the following predefined criteria. Regarding participants, studies were eligible if they enrolled pediatric patients of any age undergoing surgical or procedural interventions requiring sedation or general anesthesia. Regarding intervention, studies were required to evaluate intranasal dexmedetomidine or intranasal esketamine. Regarding outcomes, studies were required to report at least one of the following: incidence of emergence delirium, Pediatric Anesthesia Emergence Delirium (PAED) scale scores, Face Legs Activity Cry Consolability (FLACC) pain scores, emergence time, parental separation anxiety, parental satisfaction, postoperative nausea or vomiting, or total adverse events. Regarding study design, randomized controlled trials, non-randomized controlled trials, and prospective observational studies with a comparator arm were all eligible for inclusion.

Studies were excluded if they were animal studies or in vitro research, published in non-English languages, did not involve intranasal administration of the study agents, or did not include a pediatric population. Conference abstracts, editorials, letters, and review articles without extractable original data were also excluded, as were studies reporting only combined sedation routes without an intranasal-specific arm, or those focusing exclusively on non-procedural or chronic sedation contexts.

Data Extraction

Independent data extraction was performed by two reviewers using a standardized framework, with discrepancies resolved by consensus or third-reviewer adjudication. Extracted data included study descriptors (author, year, design, sample size, follow-up), participant demographics (age, sex, weight), intervention parameters (agent, dose, route), procedural characteristics (surgery type, anesthesia, setting), and baseline anxiety scores where reported. Outcome data extracted for both groups included incidence of emergence delirium, PAED scale scores, FLACC pain scores, emergence time, parental separation anxiety, parental satisfaction, postoperative nausea or vomiting (PONV), and total adverse events. For dichotomous outcomes, event counts and group totals were recorded; for continuous outcomes, means and standard deviations were extracted directly or estimated from median and interquartile range using established conversion methods where required.

Risk of Bias Assessment

The risk of bias was assessed independently by two reviewers, with disagreements resolved through discussion or third-reviewer adjudication. For randomized controlled trials, the Cochrane Risk of Bias tool version 2 (RoB 2) was used to evaluate five domains: randomization process, deviations from intended interventions, missing outcome data, outcome measurement, and selection of reported results. Each domain was judged as low risk, some concerns, or high risk, with an overall judgment assigned accordingly [12]. For the prospective cohort study, the Newcastle-Ottawa Scale (NOS) was used to assess three domains: selection of study groups, comparability, and ascertainment of outcome. Overall quality was categorized as poor, fair, or good based on the total NOS score [13]. The certainty of evidence for each outcome was assessed using the Grading of Recommendations Assessment, Development and Evaluation (GRADE) approach. Summary of findings tables were generated using GRADEpro Guideline Development Tool.

Statistical Analysis

Statistical analyses were conducted using Review Manager (RevMan) version 5.4 (Revman International, Inc., New York City, New York), with p-value <0.05 considered statistically significant. Risk ratios (RRs) with 95% CIs were calculated for dichotomous outcomes, and mean differences (MDs) with 95% CIs for continuous outcomes. Between-study heterogeneity was assessed using the I² statistic and chi-square test, with heterogeneity considered significant when I² exceeded 50% or the chi-squared p-value was less than 0.1. A fixed-effects model was applied when the data were homogeneous, and a random-effects model was used when significant heterogeneity was present.

Results

Study Selection

**Figure 1:**
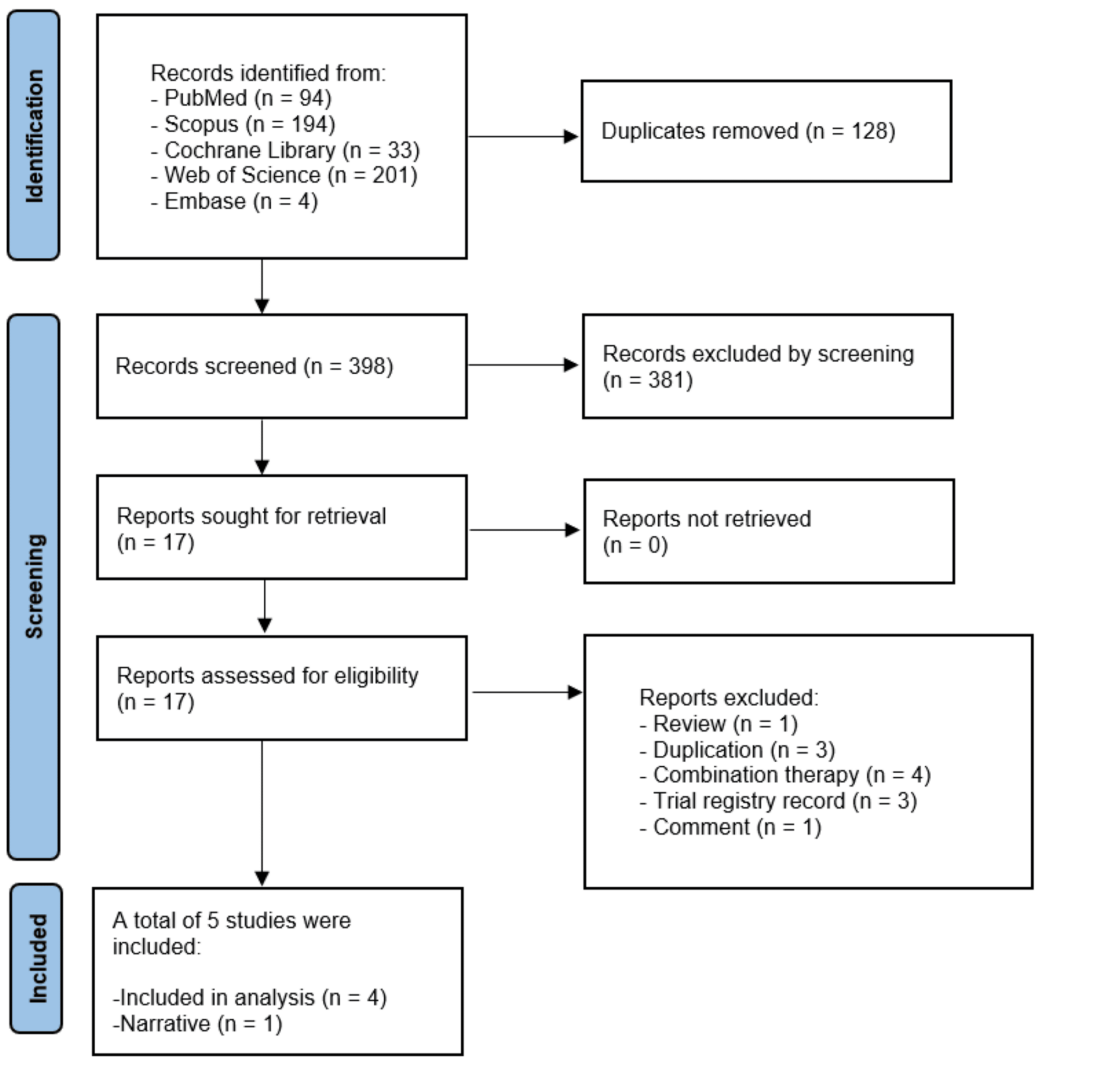
PRISMA flow diagram PRISMA - Preferred Reporting Items for Systematic Reviews and Meta-Analyses

A search of five databases yielded 526 records in total: PubMed (n=94), Scopus (n=194), Web of Science (n=201), Cochrane Library (n=33), and Embase (n=4). Following the removal of the duplicates, 398 unique records remained for title and abstract screening. The full texts of the remaining 17 reports were retrieved and assessed for eligibility. Of these, 12 were excluded for several reasons. Ultimately, five studies were included: four were incorporated into the quantitative meta-analysis [9,14-16], and one was included in the narrative synthesis only [17]. The study selection process is illustrated in Figure 1.

Characteristics of the Included Studies

Participant ages ranged from young toddlers to older children, with mean ages between 1.9 and 9.0 years across studies. Sex distribution and body weight were broadly comparable between treatment arms within each study. Preoperative anxiety scores were reported in two studies, indicating mild to moderate baseline anxiety. Baseline demographic and procedural characteristics were well-balanced across groups in all included studies (Table 1).

**Table 1:** Baseline characteristics of study participants ASA - American Society of Anesthesiologists physical status classification; GA - general anesthesia; IN - intranasal; IQR - interquartile range; m-YPAS - modified Yale Preoperative Anxiety scale; NR - not reported; SD - standard deviation

Study ID	Study Arm	n	Age (years)	Male, n (%)	Weight (kg)	ASA I, n (%)	m-YPAS score	Surgery duration (min)	Anesthesia duration (min)	Procedure
Liao et al., 2024 (9)	IN DEX 2 µg/kg	64	3.8 ± 0.9	34 (53.1%)	18.2 ± 2.6	63 (98.4%)	34.8 (24.3-43.3)	29.5 (23.0-36.8)	43.0 )36.3-52.0)	Adenotonsillectomy: 56 (87.5%); Adenoidectomy: 8 (12.5%)
	IN esketamine 1 mg/kg	63	3.7 ± 0.7	36 (57.1%)	19.0 ± 2.2	62 (98.4%)	33.3 (23.3-42.7)	28.0 (23.0-35.0)	41.0 (35.0-49.0)	Adenotonsillectomy: 52 (82.5%); Adenoidectomy: 11 (17.5%)
	IN DEX 1 µg/kg + esketamine 0.5 mg/kg (Combination)	64	3.8 ± 0.7	29 (45.3%)	18.8 ± 2.2	62 (96.9%)	32.5 (23.3-41.7)	27.5 (22.0-35.0)	40.5 (35.3-50.0)	Adenotonsillectomy: 57 (89.1%); Adenoidectomy: 7 (10.9%)
Lu et al., 2022 (14)	Group D: IN DEX 2 µg/kg	30	3.5 ± 1.6	24 (80.0%)	15.3 ± 4.0	NR	52.5 (45.4-67.0)	25.5 (14.8-32.0)	35.5 (26.3-48.5)	Lower abdominal/perineal surgery
	Group S: IN esketamine 1 mg/kg	29	2.8 ± 1.4	28 (96.6%)	15.1 ± 4.6	NR	58.2 (48.3-73.2)	25.0 (16.5-42.5)	37.0 (27.0-52.0)	Lower abdominal/perineal surgery
	Group DS: IN DEX 1 µg/kg + esketamine 0.5 mg/kg	29	3.4 ± 1.6	27 (93.1%)	16.8 ± 4.5	NR	50.0 (46.6-69.1)	23.0 (16.0-38.5)	34.0 (30.0-56.0)	Lower abdominal/perineal surgery
Nikula et al., 2024 (15)	IN dexmedetomidine 2 µg/kg	15	mean 2.2	8 (53.3%)	mean 12.9	NR	NR	NR	NR	Laceration: 12; Burn: 3; ED setting; local anesthesia
	IN esketamine 1 mg/kg	15	mean 1.9	11 (73.3%)	mean 12.4	NR	NR	NR	NR	Laceration: 15; Burn: 0; ED setting; local anesthesia
Xie et al., 2025 (16)	Control (normal saline)	41	3.8 ± 1.6	26 (63.4%)	NR	NR	NR	15.5 (6-56)‡	49.8 ± 17.9	Fiber bronchoscopy; sevoflurane + propofol induction
	IN esketamine 1 mg/kg	42	3.6 ± 1.7	28 (66.7%)	NR	NR	NR	13.2 (4-23)‡	46.9 ± 20.8	Fiber bronchoscopy; sevoflurane + propofol induction
	IN dexmedetomidine 1 µg/kg	43	3.6 ± 1.8	21 (48.8%)	NR	NR	NR	15.3 (5-41)‡	54.0 ± 22.5	Fiber bronchoscopy; sevoflurane + propofol induction
Xin et al., 2021 (17)	D1: IN DEX 1 µg/kg	37	8.1 ± 1.8	19 (51.4%)	17.6 ± 4.4	NR	NR	NR	NR	Dental surgery under GA; fearful/uncooperative patients
	D2: IN DEX 1.5 µg/kg	41	8.7 ± 1.7	20 (48.8%)	18.1 ± 5.2	NR	NR	NR	NR	Dental surgery under GA; fearful/uncooperative patients
	D3: IN DEX 2 µg/kg	34	9.0 ± 1.6	17 (50.0%)	17.9 ± 4.7	NR	NR	NR	NR	Dental surgery under GA; fearful/uncooperative patients
	K: IN esketamine 0.5 mg/kg	38	8.0 ± 1.8	20 (52.6%)	17.5 ± 4.9	NR	NR	NR	NR	Dental surgery under GA; fearful/uncooperative patients

Five studies involving 481 participants were included, comprising four RCTs and one prospective cohort study. Four studies were conducted in China and one in Sweden. The studies covered a variety of pediatric surgical and procedural contexts, including tonsillectomy, adenoidectomy, elective abdominal surgery, emergency department procedures, fiber bronchoscopy, and dental surgery. Dexmedetomidine doses ranged from 1 to 2 µg/kg and esketamine doses from 0.5 to 1 mg/kg across the included studies. Sevoflurane-based general anesthesia was the primary anesthetic in four studies, while one used local anesthesia in an emergency department setting (Table 2).

**Table 2:** Summary of the included studies CAPD - Cornell Assessment of Pediatric Delirium; DEX - dexmedetomidine; ED - emergence delirium; ESK - esketamine; FLACC - Face Legs Activity Cry Consolability; GA - general anesthesia; ICC - Induction Compliance Checklist; IN - intranasal; PACU - post-anesthesia care unit; PAED - Pediatric Anesthesia Emergence Delirium scale; PNBC - postoperative negative behavioral changes; RCT - randomized controlled trial

Study ID	Study design	Country	Procedure / setting	Study arms (interventions)	Primary anesthesia	Follow-up duration	Primary endpoint
Liao et al., 2024 [9]	RCT, prospective, double-blind, 3-arm parallel	China	Tonsillectomy and/or adenoidectomy	1) IN dexmedetomidine (DEX) 2 µg/kg; 2) IN esketamine (ESK) 1 mg/kg; 3) IN DEX 1 µg/kg + ESK 0.5 mg/kg (Combination)	Sevoflurane	7 days (PNBC); immediate (ED)	Incidence of ED (PAED scale ≥10)
Lu et al., 2022 [14]	RCT, double-blind, 3-arm parallel	China	Elective lower abdominal or perineal surgery (<2 h)	1) IN DEX 2 µg/kg (group D); 2) IN esketamine 1 mg/kg (group S); 3) IN DEX 1 µg/kg + ESK 0.5 mg/kg (group DS)	Sevoflurane	Immediate post-PACU	Induction Compliance Checklist (ICC) score
Nikula 2024 [15]	RCT, double-blind, 2-arm parallel (superiority trial)	Sweden	Minor injury procedures in the Emergency Department (laceration repair or burn wound dressing)	1) IN dexmedetomidine 2 µg/kg; 2) IN esketamine 1 mg/kg	Local anesthesia (buffered lidocaine)	Until Ramsay score 1 (full recovery)	Highest FLACC pain score during procedure
Xie et al., 2025 [16]	RCT, triple-blind, 3-arm parallel (placebo-controlled)	China	Pediatric fiber bronchoscopy (diagnostic/therapeutic)	1) IN esketamine 1 mg/kg; 2) IN dexmedetomidine 1 µg/kg; 3) Normal saline (control)	Sevoflurane + propofol induction	PACU discharge	Incidence of ED (CAPD score ≥9)
Xin et al., 2021 [17]	Prospective single-center cohort study	China	Pediatric dental surgery under GA (fearful/uncooperative patients)	1) IN DEX 1 µg/kg (D1); 2) IN DEX 1.5 µg/kg (D2); 3) IN DEX 2 µg/kg (D3); 4) IN esketamine 0.5 mg/kg (K)	General anesthesia (inhalation)	Recovery period	Sedation level (adequate/inadequate) and overall sedation success rate

Quality Assessment

**Figure 2:**
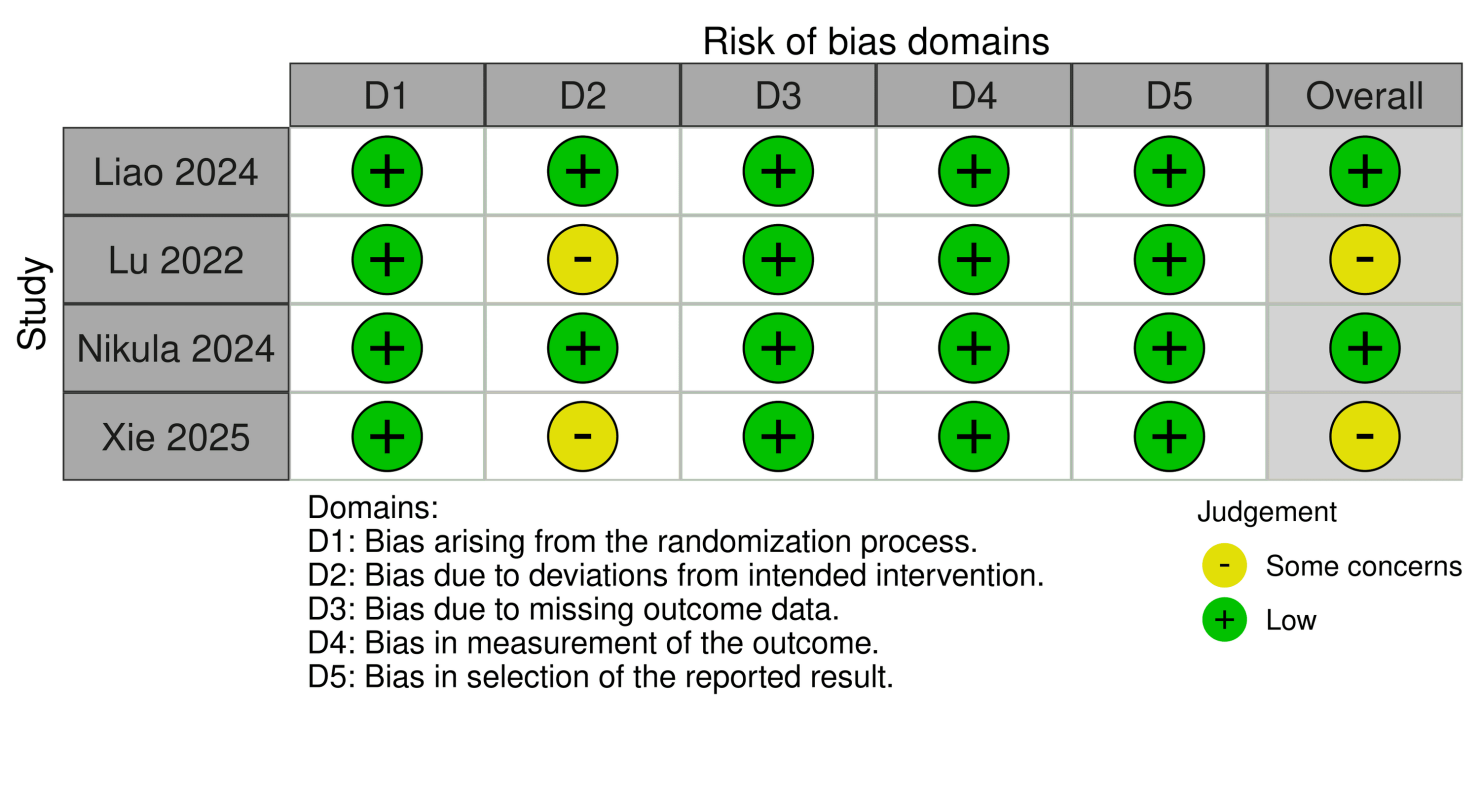
Quality assessment for randomized controlled trials (RoB2) References: [9,14–16]

For RCTs, risk of bias was assessed using the RoB 2 tool across four RCTs. Of the four studies, two were rated as low risk across all five domains, resulting in an overall low-risk-of-bias judgment. The risk-of-bias summary is presented in Figure 2.

For the cohort study, Xin et al.'s 2021 study was assessed using the Newcastle-Ottawa Scale and received a score of 8 out of 9 stars across the three domains of selection, comparability, and outcome assessment, corresponding to a good-quality rating. Full details in Table 3.

**Table 3:** Quality assessment of the included cohort studies using the Newcastle Ottawa scale D1 - Is the case definition adequate/Representative of the exposed cohort?; D2 - Representative of the cases/Selection of the non-exposed cohort; D3 - Selection of Controls/Ascertainment of exposure; D4 - Definition of Controls/ Demonstration that outcome of interest was not present at start of study; D5 - Ascertainment of exposure/ Assessment of outcome; D6 - Same method of ascertainment for cases and controls/ Was follow-up long enough for outcomes to occur; D7 - Non-Response rate/ Adequacy of follow-up of cohorts.

Study ID	Selection				Comparability	Outcome			Quality score
	D1	D2	D3	D4		D5	D6	D7	
Xin et al., 2021 [17]	*	*	*	*	*	*	*	*	Good

Primary Outcomes

**Figure 3:**
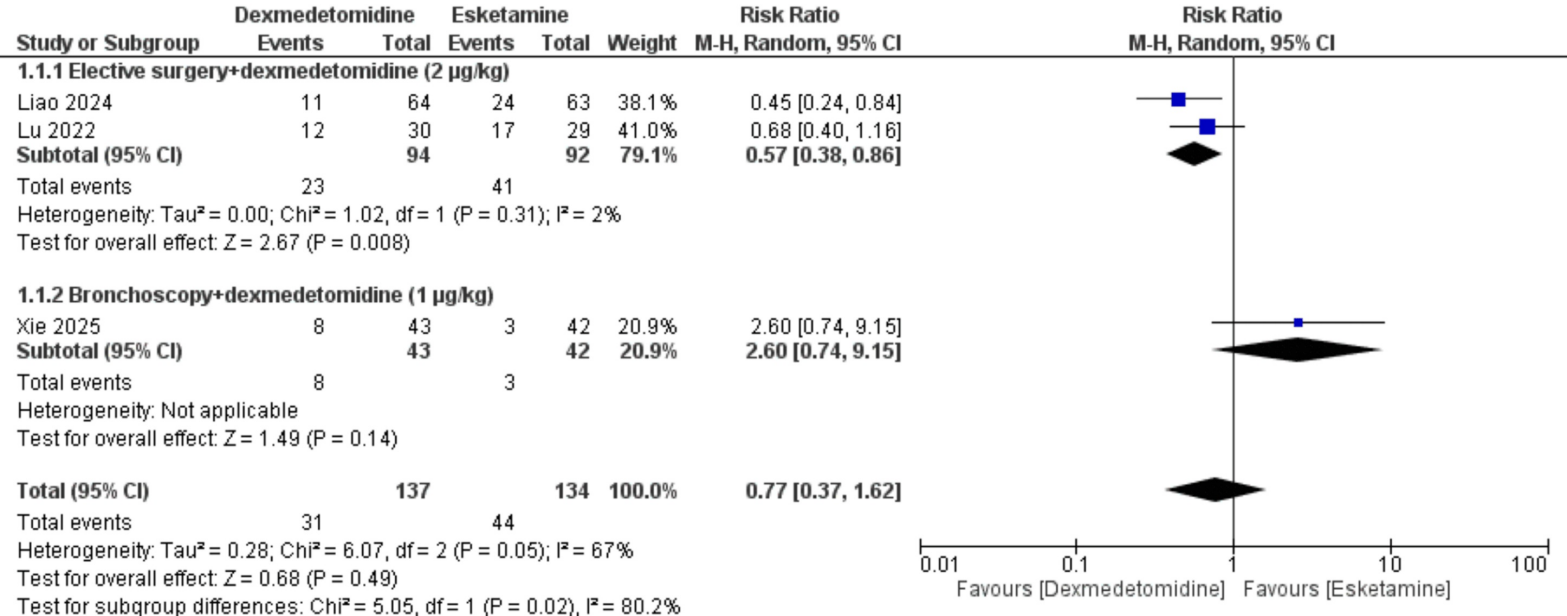
Emergence delirium References: [9,14,16]

Emergence delirium: Four studies (n=271) reported the incidence of ED. A meta-analysis with subgroup analyses by dexmedetomidine dose and clinical setting was performed. In the elective surgery subgroup, where dexmedetomidine was used at 2 µg/kg, dexmedetomidine significantly reduced ED incidence compared to esketamine (RR 0.57 (0.38, 0.86); p=0.008), with low heterogeneity (I²=2%). In the bronchoscopy subgroup where dexmedetomidine was used at the lower dose of 1 µg/kg, the effect was reversed, with esketamine showing a numerically lower ED incidence, though this did not reach statistical significance (RR 2.60 (0.74, 9.15); p=0.14). The overall pooled estimate across all studies was non-significant (RR 0.77 (0.37, 1.62); p=0.49), with substantial heterogeneity (I²=67%; Figure 3).

**Figure 4:**
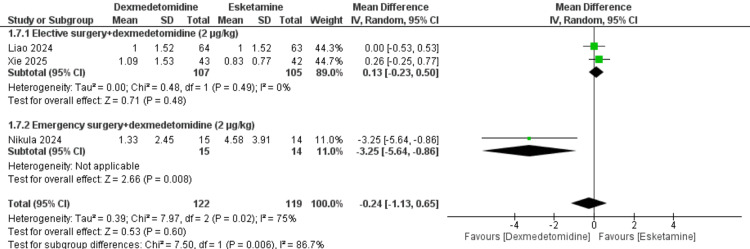
FLACC pain score FLACC - Face Legs Activity Cry Consolability References: [9,15,16]

FLACC pain score:* *Three studies (n=241) reported FLACC pain scores. Significant subgroup differences were observed (I²=86.7%; p=0.006). In the elective and bronchoscopy surgery subgroup (dexmedetomidine 2 µg/kg), no significant difference was found between the two agents (MD 0.13, 95% CI (-0.23, 0.50); p=0.48; I²=0%). However, in the emergency department subgroup, dexmedetomidine was associated with significantly lower pain scores compared to esketamine (MD -3.25, 95% CI (-5.64, -0.86); p=0.008). The overall pooled estimate was non-significant (MD -0.24, 95% CI (-1.13, 0.65); p=0.60), with high heterogeneity (I²=75%; Figure 4).

**Figure 5:**
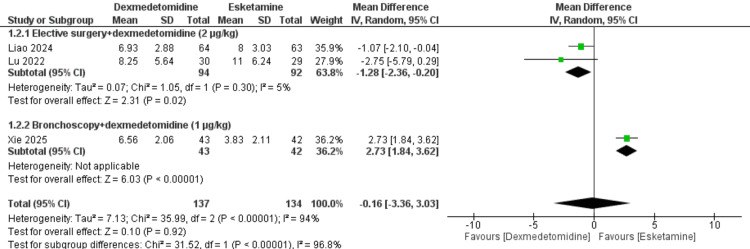
Pediatric anesthesia emergence delirium scale References: [9,14,16]

Secondary Outcomes:

Pediatric Anesthesia Emergence Delirium scale:* *Three studies (n=271) reported PAED scale scores. In the elective surgery subgroup (dexmedetomidine 2 µg/kg), dexmedetomidine was associated with significantly lower PAED scores compared to esketamine (MD -1.28 (-2.36, -0.20); p=0.02), with low heterogeneity (I²=5%). Conversely, in the bronchoscopy subgroup (dexmedetomidine 1 µg/kg), Xie et al. reported significantly higher PAED scores in the dexmedetomidine group than in the esketamine group (MD 2.73 (1.84, 3.62); p<0.00001). The overall pooled estimate was non-significant (MD -0.16 (-3.36, 3.03); p=0.92), with very high heterogeneity (I²=94%; Figure 5).

**Figure 6:**
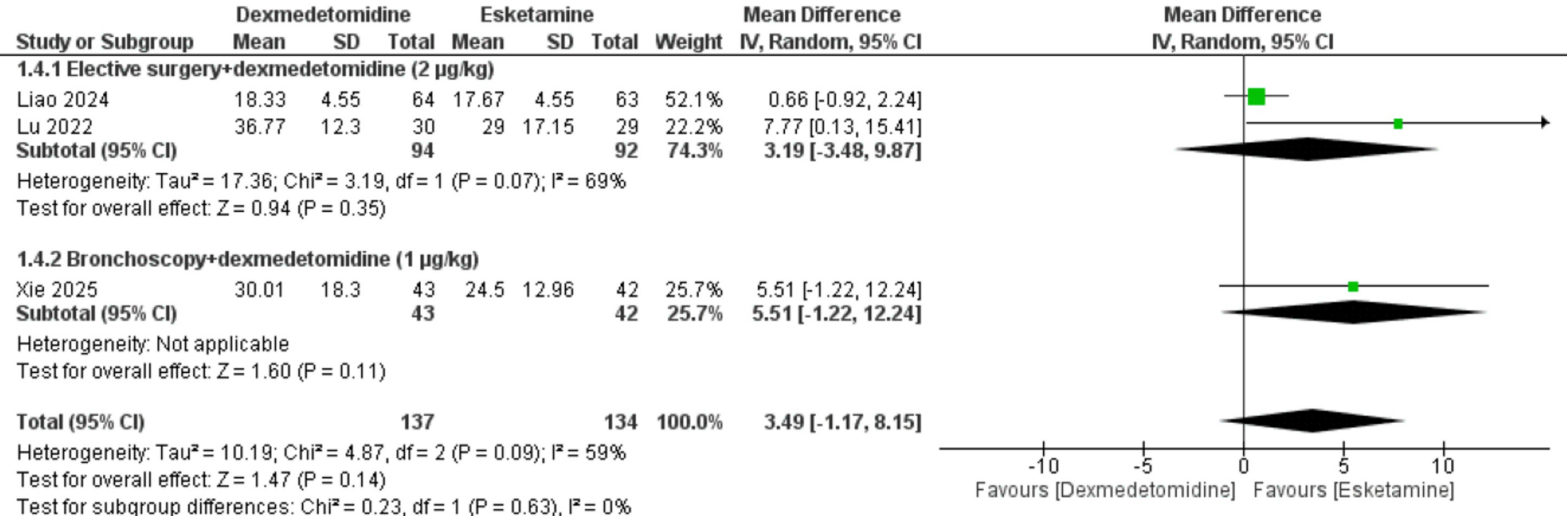
Emergence time (min) References: [9,14,16]

Emergence time (min):* *Three studies (n=271) reported emergence time in minutes. In the elective surgery subgroup, emergence time was numerically longer with dexmedetomidine compared to esketamine but did not reach statistical significance (MD 3.19, 95% CI (-3.48, 9.87); p=0.35). In the bronchoscopy subgroup, a similar non-significant trend was observed (MD 5.51, 95% CI (-1.22, 12.24); p=0.11). The overall pooled estimate was non-significant (MD 3.49, 95% CI (-1.17, 8.15); p=0.14; Figure 6).

**Figure 7:**
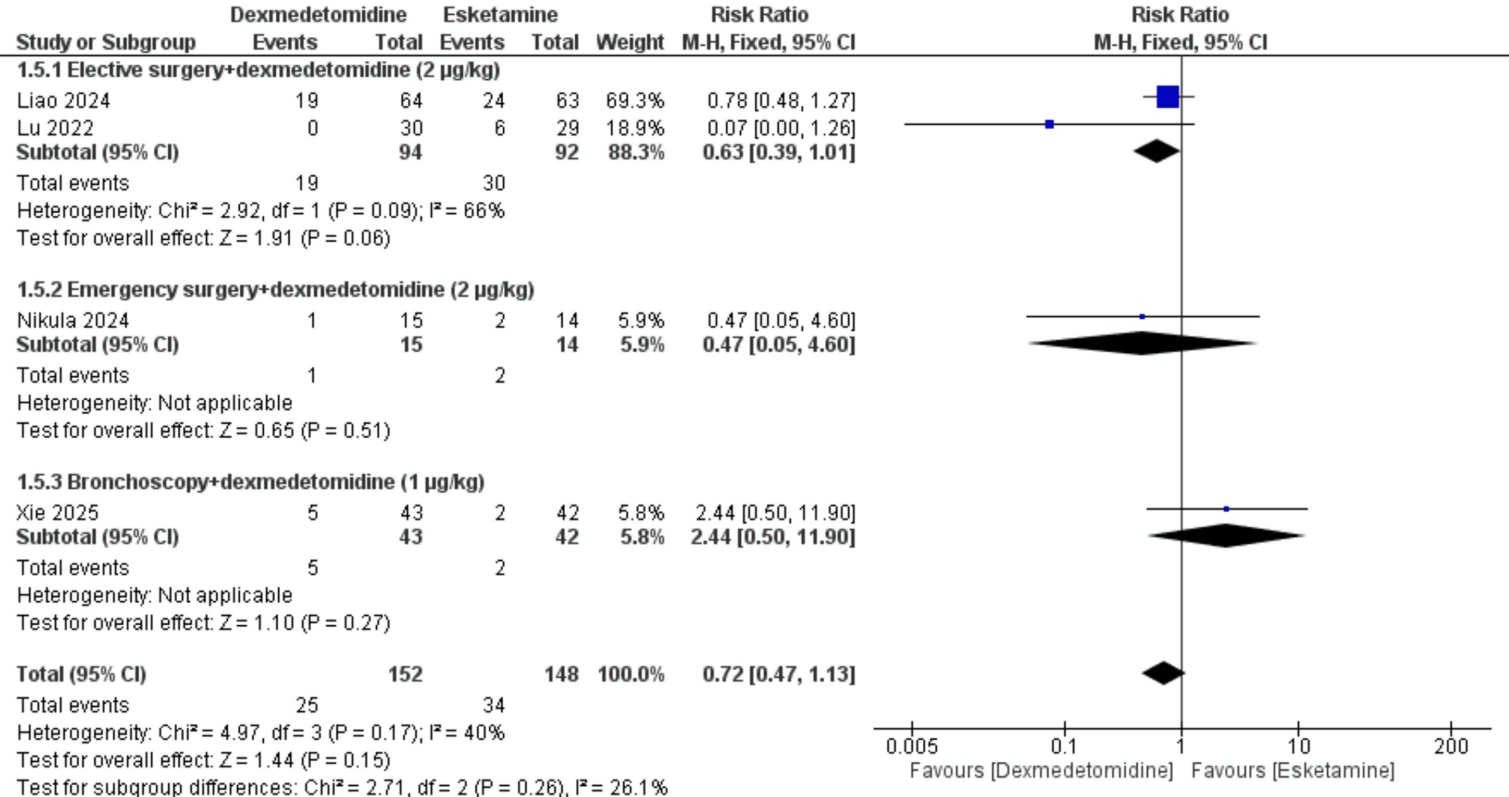
Total adverse events References: [9,14-16]

Total adverse events:* *Four studies (n=300) reported total adverse events, stratified by clinical setting. In the elective surgery subgroup (dexmedetomidine 2 µg/kg), the pooled RR approached was not significant (RR 0.63 (0.39, 1.01); p=0.06). In both the emergency department and bronchoscopy subgroups, no significant differences were observed. The overall pooled estimate was non-significant (RR 0.72 (0.47, 1.13); p=0.15; Figure 7).

**Figure 8:**
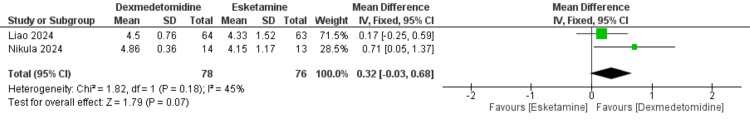
Parental satisfaction References: [9,15]

Parental satisfaction:* *Two studies (n=154) reported parental satisfaction scores. The pooled estimate favored dexmedetomidine, though not statistically significant (MD 0.32 (-0.03, 0.68); p=0.07; Figure 8).

**Figure 9:**
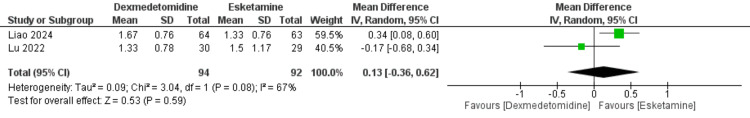
Parental separation anxiety References: [9,14]

Parental separation anxiety:* *Two studies (n=186) reported parental separation anxiety scores. No significant difference was detected between dexmedetomidine and esketamine (MD 0.13 (-0.36, 0.62); p=0.59), with high heterogeneity (I²=67%; Figure 9).

**Figure 10:**
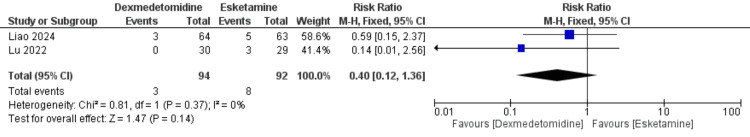
Postoperative nausea or vomiting References: [9,14]

Postoperative nausea or vomiting: Two studies (n=186) reported PONV. The incidence was numerically lower with dexmedetomidine, but the difference was not statistically significant (RR 0.40 (0.12, 1.36); p=0.14), with no heterogeneity (I²=0%; Figure 10).

Narrative Synthesis

The prospective cohort study by Xin and colleagues evaluated four intranasal premedication regimens in 150 uncooperative pediatric dental patients aged 3 to 10 years: dexmedetomidine 1 µg/kg (D1), 1.5 µg/kg (D2), 2 µg/kg (D3), and esketamine 0.5 mg/kg (K). This study was included in the narrative synthesis only, given its non-randomized cohort design and the lower esketamine dose used compared with the trials included in the quantitative analyses. Sedation onset time was significantly shorter with esketamine and with the highest dexmedetomidine dose (D3) compared with the lower dexmedetomidine doses, while recovery time was fastest in the D1 group. Intra- and postoperative pain scores were significantly lower in both the esketamine and D3 groups than in the D1 group, with esketamine showing a slight postoperative analgesic advantage over D1. Overall sedation success rates were highest in the D2 and D3 groups, followed by the D1 and esketamine groups, although these differences did not reach statistical significance. Hemodynamically, esketamine was associated with higher pulse rate and systolic blood pressure compared with all dexmedetomidine doses, consistent with its sympathomimetic profile, whereas oxygen saturation, respiratory rate, and diastolic blood pressure remained stable and comparable across all four groups. The authors concluded that both intranasal dexmedetomidine and esketamine are effective for producing moderate sedation in this population, with the higher dexmedetomidine dose (2 µg/kg) offering the most favorable balance of sedation depth, analgesia, and procedural success. These findings are broadly consistent with the dose-dependent pattern observed in the quantitative meta-analysis, particularly the apparent superiority of higher-dose intranasal dexmedetomidine in elective procedural settings, and support the inclusion of this study as supplementary narrative evidence [17].

Discussion

This systematic review and meta-analysis synthesized evidence from five studies comparing intranasal dexmedetomidine and intranasal esketamine as premedication in pediatric surgical patients. Neither agent demonstrated statistically significant pooled superiority across primary or secondary outcomes. However, subgroup analyses revealed a consistent context- and dose-dependent pattern, with dexmedetomidine at 2 µg/kg showing advantages in elective surgical settings, while esketamine appeared more favorable in bronchoscopy procedures. Adverse event profiles were broadly comparable between agents.

These findings align with two relevant prior systematic reviews. Dwivedi et al. conducted a meta-analysis comparing intranasal ketamine versus intranasal dexmedetomidine for pediatric premedication, concluding that neither agent demonstrated superiority in parental separation, mask acceptance, or intravenous cannulation, with clinical decision-making guided primarily by differences in safety profiles [18]. Another meta-analysis comparing intranasal esketamine combined with dexmedetomidine versus dexmedetomidine alone, demonstrating that the combination significantly reduced the incidence of emergence delirium and bradycardia without increasing nausea and vomiting [19]. This synergistic effect likely reflects the complementary pharmacological profiles of the two agents, whereby esketamine's sympathomimetic properties offset dexmedetomidine's bradycardic tendency while dexmedetomidine attenuates esketamine's psychomimetic side effects.

The pharmacological basis for the differential performance observed across subgroups is well-established. Dexmedetomidine produces sustained sedation and anxiolysis through α-2 adrenoceptor activation in the locus coeruleus, dampening sympathetic outflow and attenuating catecholamine release - mechanisms that likely underlie its superior suppression of emergence delirium during longer elective procedures [20,21]. This dose-dependent effect is supported by pharmacokinetic evidence that 2 µg/kg intranasal dexmedetomidine achieves targeted sedative concentrations that persist for up to two hours, whereas the 1 µg/kg dose used in the bronchoscopy subgroup appears insufficient to maintain this effect [22]. Esketamine's NMDA receptor antagonism confers rapid sedation, analgesia, and bronchodilation with preserved airway reflexes, making it particularly advantageous in shorter, airway-sensitive procedures such as bronchoscopy [23,24].

Its analgesic properties independently reduce postoperative pain, a recognized contributor to emergence delirium, which may further explain its favorable performance in that setting [15]. Of note, contrasting reports suggest that near-anesthetic intravenous doses of esketamine may paradoxically increase emergence delirium risk in preschool children, highlighting that route and dosing magnitude critically determine the safety profile of this agent [25]. The sympathomimetic cardiovascular effects of esketamine simultaneously serve as a physiological counterbalance to dexmedetomidine's bradycardic tendency, providing a strong pharmacological rationale for combination strategies [9,19].

This review has several strengths. It is the first meta-analysis to comprehensively compare these two agents as monotherapy across diverse pediatric procedural settings, with subgroup analyses by clinical context and dexmedetomidine dose revealing clinically meaningful differential patterns that pooled estimates alone obscure. Risk of bias was low to moderate across the included studies, with most using the RCT design, the gold standard for evidence. However, limitations include the small number of included studies, substantial heterogeneity in primary outcomes driven by clinical setting and dosing variability, and the geographic concentration of studies, primarily in China, which limits generalizability. Future large-scale multicenter randomized trials with standardized dosing protocols, validated outcome instruments, broader procedural representation, and longer-term behavioral follow-up are needed to establish definitive evidence-based premedication recommendations across pediatric surgical settings.

## Conclusions

This systematic review and meta-analysis suggest that intranasal dexmedetomidine and esketamine may offer broadly similar overall performance as pediatric premedication agents, although the small number of included studies and substantial heterogeneity preclude firm conclusions of equivalence. Pre-specified subgroup analyses indicated possible context- and dose-dependent differences. Dexmedetomidine at the higher dose appeared associated with lower emergence delirium incidence and PAED scores in elective surgical settings, and showed a signal of greater analgesic benefit in the emergency department setting. Conversely, in the bronchoscopy setting using a lower dose of dexmedetomidine, esketamine was associated with lower PAED scores, suggesting that dose-response and procedural context are likely interrelated. Although adverse event rates and emergence times did not differ significantly between agents, the limited sample size and inability to pool individual adverse events preclude firm conclusions regarding comparative safety. The high heterogeneity observed in overall pooled estimates underscores that these findings are best regarded as hypothesis-generating rather than practice-changing. Given the small evidence base, predominantly single-region origin of included studies, and wide confidence intervals across most outcomes, the present results are insufficient to recommend one agent over the other. Agent selection should continue to be guided by clinician judgment, regulatory approval status, institutional protocols, and patient-specific factors. Future research should prioritize adequately powered, geographically diverse multicenter trials with standardized dosing, head-to-head comparisons against current standards of care, longer-term behavioral outcome assessment, and evaluation of combination intranasal regimens, given the pharmacological complementarity of the two agents.

## Appendices

**Table 4:** Advanced search strategy

Database	Search Query	Results
PubMed	(Dexmedetomidine OR MPV-1440 OR MPV1440 OR "MPV 1440" OR MED-001 OR Precedex OR Igalmi OR Sedadex OR Dexdor OR "D-medetomidine" OR "S-medetomidine" OR "S(+)-medetomidine") AND (Esketamine OR "L-Ketamine" OR "(-)-Ketamine" OR "S-Ketamine" OR "S(+)-ketamine" OR dextroketamine OR JNJ-54135419 OR Kataved OR Ketanest OR Spravato) AND (Intranasal* OR Intra-nasal OR Nasal OR Transnasal OR Trans-nasal OR Endonasal OR Rhinonasal OR Nose OR Nebulize*) AND (pediatr* OR child* OR adolescen* OR paediatr* OR infan* OR Toddler OR Juvenile OR Minor* OR Youth OR Teen OR School-age OR "Young person" OR "Young people")	94
Web of Science (WOS)	TS=((Dexmedetomidine OR MPV-1440 OR MPV1440 OR “MPV 1440” OR MED-001 OR Precedex OR Igalmi OR Sedadex OR Dexdor OR “D-medetomidine” OR “S-medetomidine” OR “S(+)-medetomidine”) AND (Esketamine OR “L-Ketamine” OR “(-)-Ketamine” OR “S-Ketamine” OR “S(+)-ketamine” OR dextroketamine OR JNJ-54135419 OR Kataved OR Ketanest OR Spravato) AND (Intranasal* OR Intra-nasal OR Nasal OR Transnasal OR Trans-nasal OR Endonasal OR Rhinonasal OR Nose OR Nebulize*) AND (pediatr* OR child* OR adolescen* OR paediatr* OR infan* OR Toddler OR Juvenile OR Minor* OR Youth OR Teen OR School-age OR “Young person” OR “Young people”))	201
Scopus	TITLE-ABS-KEY ( ( Dexmedetomidine OR MPV-1440 OR MPV1440 OR "MPV 1440" OR MED-001 OR Precedex OR Igalmi OR Sedadex OR Dexdor OR "D-medetomidine" OR "S-medetomidine" OR "S(+)-medetomidine" ) AND ( Esketamine OR "L-Ketamine" OR "(-)-Ketamine" OR "S-Ketamine" OR "S(+)-ketamine" OR dextroketamine OR JNJ-54135419 OR Kataved OR Ketanest OR Spravato ) AND ( Intranasal* OR Intra-nasal OR Nasal OR Transnasal OR Trans-nasal OR Endonasal OR Rhinonasal OR Nose OR Nebulize* ) AND ( pediatr* OR child* OR adolescen* OR paediatr* OR infan* OR Toddler OR Juvenile OR Minor* OR Youth OR Teen OR School-age OR "Young person" OR "Young people" ) )	194

**Table 5:** GRADE summary of findings table CI - confidence interval; FLACC - Face Legs Activity Cry Consolability scale; MD - mean difference; PAED - Pediatric Anesthesia Emergence Delirium scale; PONV - postoperative nausea or vomiting; RR - risk ratio a) Downgraded one level for serious inconsistency: substantial heterogeneity detected (I² >50%) across included studies; b). Downgraded one level for serious imprecision: wide confidence interval crossing the line of no effect with limited sample size.

Certainty assessment							№ of patients		Effect		Certainty	Importance
№ of studies	Study design	Risk of bias	Inconsistency	Indirectness	Imprecision	Other considerations	Intranasal dexmedetomidine	Intranasal esketamine	Relative (95% CI)	Absolute (95% CI)		
Emergence delirium												
3	randomised trials	not serious	serious^a^	not serious	not serious	none	31/137 (22.6%)	44/134 (32.8%)	RR 0.77 (0.37 to 1.62)	76 fewer per 1,000 (from 207 fewer to 204 more)	⨁⨁⨁◯ Moderate^a^	CRITICAL
Paediatric anaesthesia emergence delirium scale												
3	randomised trials	not serious	serious^a^	not serious	serious^b^	none	137	134	-	MD 0.16 lower (3.36 lower to 3.03 higher)	⨁⨁◯◯ Low^a,b^	IMPORTANT
Parental separation anxiety												
2	randomised trials	not serious	serious^a^	not serious	not serious	none	94	92	-	MD 0.13 higher (0.36 lower to 0.62 higher)	⨁⨁⨁◯ Moderate^a^	IMPORTANT
Emergence time (min)												
3	randomised trials	not serious	serious^a^	not serious	not serious	none	137	134	-	MD 3.49 higher (1.17 lower to 8.15 higher)	⨁⨁⨁◯ Moderate^a^	IMPORTANT
Total adverse events												
4	randomised trials	not serious	not serious	not serious	not serious	none	25/152 (16.4%)	34/148 (23.0%)	RR 0.72 (0.47 to 1.13)	64 fewer per 1,000 (from 122 fewer to 30 more)	⨁⨁⨁⨁ High	IMPORTANT
Postoperative nausea or vomiting												
2	randomised trials	not serious	not serious	not serious	not serious	none	3/94 (3.2%)	8/92 (8.7%)	RR 0.40 (0.12 to 1.36)	52 fewer per 1,000 (from 77 fewer to 31 more)	⨁⨁⨁⨁ High	IMPORTANT
FLACC pain score												
3	randomised trials	not serious	serious^a^	not serious	not serious	none	122	119	-	MD 0.24 lower (1.13 lower to 0.65 higher)	⨁⨁⨁◯ Moderate^a^	CRITICAL
Parental satisfaction												
2	randomised trials	not serious	not serious	not serious	not serious	none	78	76	-	MD 0.32 higher (0.03 lower to 0.68 higher)	⨁⨁⨁⨁ High	IMPORTANT
